# Loss of cyclin‐dependent kinase‐like 2 predicts poor prognosis in gastric cancer, and its overexpression suppresses cells growth and invasion

**DOI:** 10.1002/cam4.1577

**Published:** 2018-05-23

**Authors:** Chia‐Lang Fang, Yih‐Huei Uen, Han‐Kun Chen, You‐Cheng Hseu, Chih‐Chan Lin, Shih‐Ting Hung, Ding‐Ping Sun, Kai‐Yuan Lin

**Affiliations:** ^1^ Department of Pathology School of Medicine College of Medicine Taipei Medical University Taipei Taiwan; ^2^ Department of Pathology Wan Fang Hospital Taipei Medical University Taipei Taiwan; ^3^ Department of Surgery Asia University Hospital Taichung Taiwan; ^4^ Department of Surgery Chi Mei Medical Center Tainan Taiwan; ^5^ Department of Cosmeceutics China Medical University Taichung Taiwan; ^6^ Department of Health and Nutrition Biotechnology Asia University Taichung Taiwan; ^7^ Department of Medical Research Chi Mei Medical Center Tainan Taiwan; ^8^ Department of Nutrition Chia Nan University of Pharmacy and Science Tainan Taiwan; ^9^ Department of Biotechnology Chia Nan University of Pharmacy and Science Tainan Taiwan

**Keywords:** CDKL2, gastric cancer, immunohistochemistry, prognosis

## Abstract

Cyclin‐dependent kinase‐like 2 (CDKL2), a new member of the cyclin‐dependent kinase family, may be involved in gastric cancer (GC) progression. Thus, we conducted this study to explore the clinical effect of CDKL2 in GC. Immunohistochemistry was used to measure CDKL2 levels in gastric tissues. The association of a high CDKL2 level with clinical and pathological characteristics, and the correlation between the CDKL2 level and disease‐free and overall survival were analyzed. Transfection was employed to overexpress CDKL2 in GC cells and to investigate the effect of CDKL2 overexpression on cell proliferation and invasion. Loss of CDKL2 was positively correlated with several clinical and pathological characteristics, and patients with a low CDKL2 level had significantly poorer disease‐free and overall survival than those with a high level (*P *=* *.005 and .001, respectively). Univariate analysis using the Cox proportional hazards model indicated that a low CDKL2 level was a prognosticator for inferior disease‐free survival (*P *=* *.007). Based on immmunoblotting data, AGS and HGC‐27 GC cells were chosen for CDKL2 overexpression. Cellular studies revealed that CDKL2 overexpression impaired cell proliferation and invasion. Loss of CDKL2 may serve as a biomarker for predicting GC patient outcomes and a potential therapeutic target for GC treatment.

## 1 INTRODUCTION

Gastric cancer (GC) represents a serious health threat. It is the fourth most common cancer and the third most common cause of cancer‐related death worldwide.^1^ The outcomes of patients with GC remain poor due to a poorly understood pathogenesis and lack of novel therapeutic options.^2^, ^3^ Therefore, identifying potential novel biomarkers may improve the prediction of relapse and metastasis and enhance the prognosis and therapeutic responsiveness of patients with GC. GC has evolved among various genetic alterations, and molecular pathology studies may provide an understanding of the molecular variables that cause GC and useful prognostic biomarkers.^4^, ^5^, ^6^, ^7^, ^8^, ^9^, ^10^

Cancer is a disease involving uncontrolled cell growth, and carcinogenesis is usually linked to a series of changes in the activity of cell growth regulators.^11^ Cell cycle progression is strictly modulated by orchestrated actions of cyclins with cyclin‐dependent kinases (CDKs).^12^ The CDK‐like (CDKL) family has similar attributes to the CDK family but is not known to bind to cyclins. It includes 5 members (CDKL1 to CDKL5) and is considered a separate branch of the CDK family.^13^ CDKL1 and CDKL2 are similar and presumably derive from an early vertebrate duplication. *cdkl2*, located on chromosome 4, was firstly cloned from a human fetal brain.^14^ Its protein product, CDKL2, accumulates primarily in the cytoplasm, with lower levels in the nucleus. Little is known about the expression and function of CDKLs. CDKL1 exists predominantly in the brain, lung, kidney, and ovary,^13^, ^14^ and its overexpression has been observed in glial cells during gliosis.^15^ Using immunohistochemistry, a study by Kim et al^16^ reported that the CDKL1 protein level was augmented during postnatal heart development in rats. CDKL3 was increased in fast‐growing (suspension) HeLa cells, and CDKL3 overexpression in slow‐growing (attached) HeLa cells promoted cell proliferation. Moreover, flow cytometric analysis has demonstrated that cells with an insert of *cdkl3* could move from the G0/G1 phases to the S phase faster than control cells.^17^, ^18^ CDKL2 also exists in various brain neurons in mice, and its expression has been reported to be induced in rabbit brains during a learning test. Knockout mice data have indicated a role for CDKL2 in cognitive function.^19^, ^20^, ^21^, ^22^ According to the publicly available Oncomine database, the CDKL2 level in nontumor tissues is higher than that in tumor tissues in all reported cancer types (including brain tumor, colorectal cancer, kidney cancer, lung cancer, and breast cancer). By contrast, one study revealed that CDKL2 was upregulated in breast cancer.^23^ The expression of CDKL2 in GC is still unknown.

The role of CDKLs in cancer progression has gained increasing attention in recent years. CDKL1 overexpressed is greater in breast cancer tissues than in benign tissues. CDKL1‐knockdown breast cancer cells were reported to be arrested at the G2/M phase and were more sensitive to cell cycle chemotherapeutic drugs.^24^ In addition, the CDKL1 level was considerably higher in GC tissues than in paired normal tissues, and CDKL1 silencing in GC cells decreased the amount of proliferating cell nuclear antigen and increased that of Bik pro‐apoptotic protein and then suppressed cell proliferation and induced apoptosis.^25^ Re‐analysis of a breast cancer GWAS study suggested that CDKL2 may contribute to cancer. Li et al demonstrated that human mammary gland epithelial cells that expressed CDKL2 had increased epithelial‐mesenchymal transition (EMT) and stem cell properties, which were obtained from the activation of a positive feedback loop comprising ZEB1, E‐cadherin, and β‐catenin. Moreover, CDKL2 promoted xenograft proliferation and metastasis in vivo. In particular, CDKL2 is overexpressed in mesenchymal breast cancer cells compared with epithelial cells, and its overexpression is negatively correlated with disease‐free survival.^23^ In summary, the described studies have revealed crucial roles of CDKLs in EMT and carcinogenesis and suggested that CDKLs could be potential biomarkers for prognosis as well as gene targets for cancer therapy. To our knowledge, the role played by CDKL2 in human GC is still unknown.

The aims of this study were to measure the CDKL2 levels in normal and GC tissues and cell lines, to evaluate the prognostic effect of CDKL2 in GC, and to study the role of CDKL2 in GC tumorigenicity.

## 2 MATERIALS AND METHODS

### 2.1 Patients and specimens

Paired GC tissues and adjacent nontumor tissues from 151 patients who underwent surgical resection between 1998 and 2011 at Wan Fang Hospital (Taipei, Taiwan) were collected. All patients with GC in this study received radical total or subtotal gastrectomy with D2 lymph node dissection, serving as a standard radical surgery for GC. Postoperatively, all patients were evaluated for the necessity of further adjuvant treatment, based on pathologic TNM staging and prognostic factors. In accordance with the standard practice guideline for GC at Wan Fang Hospital, in this study, patients with T3 or T4 tumors and nodal status of N2 or N3 received postoperative adjuvant chemotherapy. Tumor and nontumor pairs of gastric tissues were analyzed for CDKL2 expression. Clinical and pathological characteristics were listed, as provided by the American Joint Committee on Cancer (AJCC) classification. Disease‐free survival was defined as the length of time after surgery during which no relapse is found, based on medical records. Surgically resected tissues from each patient were used to examine CDKL2 levels. All patients provided written informed consents, and the study was approved by the Institutional Review Board of Wan Fang Hospital (Approval No. 99049). We confirm that all experiments were performed in keeping with the relevant guidelines and regulations.

### 2.2 Immunohistochemistry

The surgical specimens of gastrectomy were fixed in neutral buffered formalin for 12‐15 hours before sampling blocks. The representative gastric tissue formalin‐fixed paraffin‐embedded blocks were used for immunohistochemistry. Five‐micrometer sections were sliced and adhered to microscope slides (catalog number: 5196, Muto Pure Chemicals, Tokyo, Japan). Positive control staining for CDKL2 was performed using a normal kidney. To retrieve antigen, deparaffinized sections were placed in sodium citrate buffer (pH, 6.0; catalog number: TA00H01, BIOTnA Biotech, Kaohsiung, Taiwan) and boiled for 40 minutes. Five percent of normal goat serum (catalog number: ab7481, Abcam, Cambridge, UK) was applied to block nonspecific staining. The sections were then incubated with the primary antibody (1:100 dilution in Antibody Diluent (catalog number: S3022, Dako, Glostrup, Denmark) of mouse monoclonal anti‐CDKL2 (catalogue number: LS‐B4479, LifeSpan BioSciences, Seattle, WA) for 2 hours at room temperature. CDKL2 staining was detected using the avidin‐biotin‐peroxidase complex protocol according to the manufacturer’s instructions (Dako REAL EnVision Detection System, catalog number: K5007, Dako). Diaminobenzidine was used for color development, and hematoxylin was used as a nuclear counterstain. The immunoreactivity of cancer cells and normal gastric glandular epithelial cells was interpreted under a light microscope (Olympus BX51) by a pathologist (CLF) who was blinded to the clinical data. For every case, five 200× fields were evaluated and scored and the average immunoreactivity was calculated and recorded on a semiquantitative scale: 0 for none, 1 for up to 25%, 2 for 25%‐50%, and 3 for >50% of the tissue showing positive staining. Sections with a score of 0 or 1 were considered to have low CDKL2 expression, and those with a score of 2 or 3 were considered to have high CDKL2 expression.

### 2.3 Cell culture

A human normal gastric cell line (Hs738.St/Int, catalog number: CRL‐7869) and 4 GC cell lines (AGS, catalog number: BCRC60102; TMC‐1, catalog number: BCRC 60379; HGC‐27, catalog number: 94042256; and 23132/87, catalog number: ACC 201) were obtained from the American Type Culture Collection (ATCC, Manassas, VA), the Bioresource Collection and Research Center (BCRC, Hsinchu, Taiwan), the European Collection of Cell Cultures (ECACC, Salisbury, UK), and Creative Bioarray (Shirley, NY), respectively. All cell lines were authenticated by the ATCC, BCRC, ECACC, and Creative Bioarray cell biology program and were not passaged for longer than 40 passages before original frozen stocks were thawed and used or a new cell aliquot was purchased. The companies performed cell line characterization using short tandem repeat profiling. Cell lines were maintained in DMEM (Hs738.St/Int; catalog number: 10569‐010, Life Technologies, Grand Island, NY), F‐12K (AGS; catalog number: 10‐025, Corning, Corning, NY), RPMI‐1640 (TMC‐1 and HGC‐27; catalog number: A1049101, Life Technologies), and MEM (23132/87; catalog number: 10‐009, Corning) with 10% fetal bovine serum (catalog number: 04‐001‐1A, Biological Industries, Cromwell CT), 100 units/mL penicillin G, 100 μg/mL streptomycin sulfate, and 250 ng/mL amphotericin B (catalog number: 15240‐062, Life Technologies).

### 2.4 Whole protein extraction

The cells were lysed in the protease/phosphatase‐containing RIPA Protein Extraction Reagent (catalog number: 89900, Pierce Biotechnology, Rockford, IL) according to the manufacturer’s protocol. The protein concentration was assayed with the BCA Protein Assay Kit (catalog number: 23225, Pierce Biotechnology), with bovine serum albumin serving as a standard. The whole proteins were frozen at −80°C before immunoblotting.

### 2.5 Immunoblotting

Ten percent SDS‐PAGE gels were used to separate proteins (30 μg/well). After electrophoresis, the separated proteins were transferred to nitrocellulose membranes (catalog number: NBA085C001EA, PerkinElmer, Waltham, MA) using a wet transfer method. The membranes were blocked with 5% nonfat milk (catalog number: sc‐2324, Santa Cruz Biotechnology, Dallas, TX) in 1× PBS (catalog number: 21‐040, Life Technologies) and incubated at 4°C overnight. The membranes were then incubated with a different mouse monoclonal anti‐CDKL2 antibody (1:100 dilution in 5% milk/0.05% 1× PBST; catalog number: H00008899‐M01; Abnova, Taipei, Taiwan) at room temperature for 1 hour. Subsequently, the peroxidase‐conjugated secondary antibodies (1:100 000 dilution in 5% milk/0.05% 1× PBST; catalog number: A4416, Sigma, Saint Louis, MO) were added to the membranes for 45 minutes at room temperature. The protein bands were detected using the Western Lighting ECL Ultra Chemiluminescence Substrate (catalog number: NEL113001EA, PerkinElmer) and analyzed with Fuji Image Gauge software (Fuji Photo Film Co., Tokyo, Japan). GAPDH acted as a protein loading control.

### 2.6 Transfection

Commercial human CDKL2 cDNA ORF or empty vectors (catalog numbers: RC510780 and PS100001, OriGene, Rockville, MD, USA) were transfected into AGS and HGC‐27 cells using TurboFect transfection reagent according to the manufacturer’s instructions (catalog number: R0531, Thermo Scientific, Waltham, MA). Briefly, the cells were seeded in a 6‐cm dish in complete medium 24 hours before transfection. The cells were then transiently transfected with 6 μg CDKL2 cDNA ORF or empty vector for 24 hours. G418 (500 μg/mL; catalog number: G8168, Sigma)‐resistant stable clones were selected. Immunoblotting was performed to evaluate the transfection efficiency.

### 2.7 Colony formation assay

Cells with a density of 500 cells per well were seeded into 6‐well plates. After 12 days postplating, individual colonies (>50 cells/colony) were fixed, stained with 1% crystal violet/methanol solution, and then scanned with a Scanjet 2200c scanner (HP, Palo Alto, CA). Next, methanol was added to solubilize the crystal violet within the cells. The absorbance was then detected at a wavelength of 540 nm to quantify the number of colonies formed. The assay was performed in triplicate, and the data are shown as the mean ± the standard deviation (SD).

### 2.8 In vitro invasion assay

The invasive capacity of the cells was determined using a Cell Invasion Assay Kit (catalog number: ECM55, Merck Millipore, Darmstadt, Germany) according to manufacturer’s protocol. Briefly, serum‐free media containing 2 × 10^5^ cells were added to ECMatrix‐layered cell culture inserts (containing polycarbonate membranes with an 8 μm pore size) after 24‐well plates were filled with complete media. The cells were then cultured for 24 hours. After incubation, the media and noninvasive cells were removed. The inserts were then dipped in the staining solution (containing crystal violet) to stain invaded cells on the lower surface of the membranes. The cultures were photographed (100× magnification, using a Leica DMIRB microscope), and the number of invaded cells was counted. The assay was conducted 3 times independently, and the results are presented as the mean ± SD.

### 2.9 Statistical analysis

Critical clinical and pathological characteristics were analyzed, which are listed as follows: age and sex of patients, Lauren classification, invasive depth of tumor, regional lymph node metastasis, distant metastasis, pathologic staging, histologic type and grade, and lymphovascular permeation. The chi‐square test was used to analyze the association between CDKL2 level and each clinical and pathological characteristic. Survival curves, namely disease‐free survival and overall survival, were plotted using the Kaplan‐Meier method, and differences in disease‐free survival were determined using the univariate log‐rank test. A statistically significant difference was defined as a *P* value of less than .05. Characteristics demonstrating significant *P* values from the univariate analysis were entered into the multivariate Cox regression model, and the hazard ratio (HR) and independence of prognostic impact were calculated in a stepwise backward fashion. A two‐tailed Student’s *t* test was used to analyze the differences in cell proliferation and invasion between CDKL2 overexpressed cells and control cells. All statistical analyses were performed using SPSS 24.0 software (IBM, New York, NY).

## 3 RESULTS

### 3.1 Basic data of patients

In total, 151 patients with GC—100 men and 51 women—were enrolled in this study (Table 1). The mean age for all patients at first diagnosis was 69.5 years (ranging from 34 to 96 years). According to the AJCC classification, 26 stage I patients, 38 stage II patients, 69 stage III patients, and 18 stage IV patients were present. The mean follow‐up time for all patients was 926 days (ranging from 5 to 3709 days). During follow‐up, 99 patients died.

**Table 1:** Demographic data and survival in different stages of GC according to the AJCC classification

	Stage I (n = 26)	Stage II (n = 38)	Stage III (n = 69)	Stage IV (n = 18)	Total (n = 151)
Gender of patients					
Male	16	25	48	11	100
Female	10	13	21	7	51
Age of patients (y)^a^	67.0 (12.1)	75.3 (10.2)	70.0 (12.8)	59.0 (14.9)	69.5 (13.1)
Follow‐up period (d)^a^	1575.0 (1142.7)	946.0 (696.5)	819.5 (822.5)	360.3 (285.8)	926.7 (877.5)
Survival					
Yes	17	18	15	2	52
No	9	20	54	16	99

a Age of patients and follow‐up period are expressed as the mean (SD).

### 3.2 Correlation between downregulation of CDKL2 and clinical and pathological characteristics in GC

**Figure 1:**
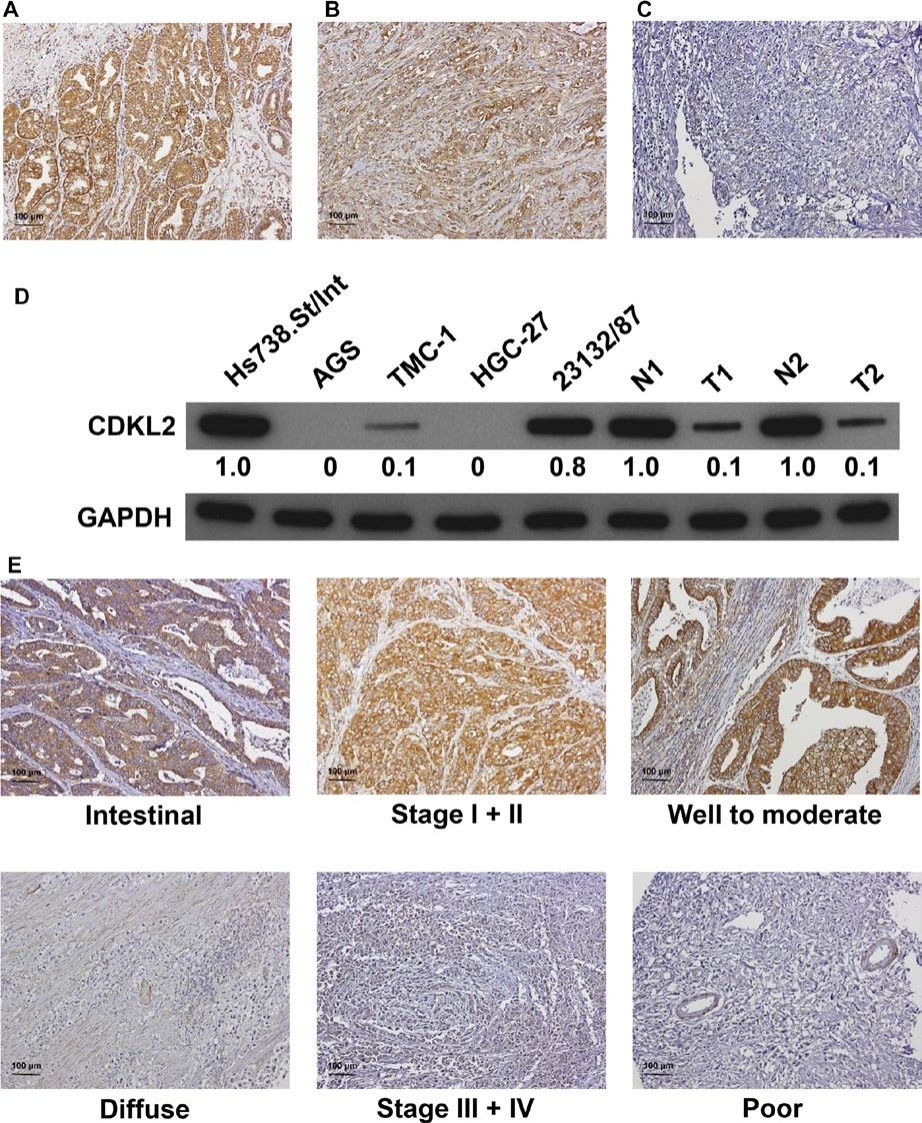
CDKL2 expression in gastric tissues and cell lines. Panels A to C. Gastric tissue specimens analyzed by immunohistochemistry with an antibody against CDKL2. Panel A shows a sample of nontumor tissue with high CDKL2 expression; Panel B shows a tumor specimen with low CDKL2 expression; Panel C shows a tumor specimen without CDKL2 expression. D, CDKL2 protein expression was examined in 5 gastric cell lines and 2 nontumor/tumor pairs of gastric tissues. N, nontumor; T, tumor. E, Representative CDKL2 staining for different parameters. The blots were first hybridized with CDKL2 antibody and, after stripping, rehybridized with β‐actin antibody. The immunoblots in the figure were cropped

Immunohistochemical analysis was employed to examine the CDKL2 level in GC tissues (Figure 1A‐C). The examined GC tissues showed negative or weak CDKL2 expression, whereas the nontumor tissues had strongly positive CDKL2 expression (*P *<* *.001). Among the 151 tumor and nontumor pairs, 68 GC tissues (45.0%) showed low CDKL2 levels (scores of 0 or 1) and 89 nontumor tissues (58.9%) showed high CDKL2 levels (scores of 2 or 3). Immunoblotting confirmed that the CDKL2 level was substantially decreased in the GC tissues and cell lines compared with normal tissues and cell lines (Figure 1D). Table 2 shows that low CDKL2 expression was correlated with Lauren classification, pathologic staging and histologic type, and grade (*P *=* *.0015, .0408, and .0001, respectively). Representative photographs of CDKL2 expression for different characteristics are shown in Figure 1E. Other clinical and pathological characteristics were found not to be significantly correlated with the CDKL2 level (Table 2).

**Table 2:** CDKL2 expression in GC and its correlation with clinical and pathological parameters

Variables	n	CDKL2 expression		*P* a
		Score = 0 or 1 (n = 83)	Score = 2 or 3 (n = 68)	
Age of patients (y)				
≥66	100	53	47	.4963
<66	51	30	21	
Gender of patients				
Male	100	53	47	.4963
Female	51	30	21	
Lauren classification				
Intestinal	102	47	55	.0015
Diffuse	49	36	13	
Invasive depth of tumor				
T1 + T2	35	16	19	.2094
T3 + T4	116	67	49	
Regional lymph node metastasis				
N0	45	21	24	.1816
N1 + N2 + N3	106	62	44	
Distant metastasis				
Absent	133	71	62	.2878
Present	18	12	6	
Pathologic staging				
I + II	64	29	35	.0408
III + IV	87	54	33	
Histologic type and grade				
Poor	68	49	19	.0001
Well to moderate	83	34	49	
Lymphovascular permeation				
Absent	44	23	21	.6966
Present	107	60	47	

a All statistical tests were two‐tailed. Significance level: *P *<* *.05.

### 3.3 Loss of CDKL2 is a poor prognosticator for GC

**Figure 2:**
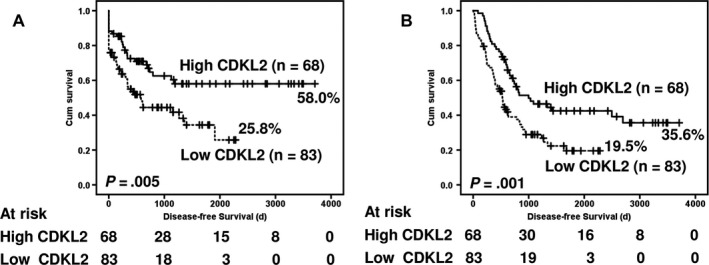
Survival analysis of patients with GC stratified by CDKL2 immunoreactivity. Panel A shows disease‐free survival. Patients with low CDKL2 expression had a 5‐y disease‐free survival rate of 34.4% compared with 58.0% for patients with high CDKL2 expression. Panel B shows overall survival. Patients with low CDKL2 expression had a 5‐y overall survival rate of 19.5% compared with 42.5% for patients with high CDKL2 expression. Two‐tailed *P *<* *.05 was considered statistically significant

The correlations of the patients’ outcomes with CDKL2 expression are shown in Figure 2. Patients with low CDKL2 expression had significantly poorer disease‐free survival and overall survival than patients with high CDKL2 expression (Figure 2A, B, *P *=* *.005 and .001, respectively). The 5‐year disease‐free survival rate of patients with low CDKL2 levels was 0.344 (95% confidence interval [CI] 0.2068 to 0.4812), whereas that of patients with high CDKL2 levels was 0.580 (95% CI 0.4506 to 0.7094). The 5‐year overall survival rate of patients with low CDKL2 levels was 0.195 (95% CI 0.0911 to 0.2989), whereas that of patients with high CDKL2 levels was 0.425 (95% CI 0.3015 to 0.5485).

Adjuvant chemotherapy was added as a variable, and data of the univariate analysis of the prognostic biomarkers of GC are summarized in Table 3. Loss of CDKL2 (*P *=* *.007), Lauren classification (*P *=* *.022), invasive depth of tumor (*P *=* *.001), regional lymph node metastasis (*P *<* *.001), distant metastasis (*P *<* *.001), pathologic staging (*P *<* *.001), histologic type and grade (*P *=* *.002), lymphovascular permeation (*P *<* *.001), and adjuvant chemotherapy (*P *<* *.001) were significantly correlated with disease‐free survival. The multivariate analysis demonstrated that only distant metastasis (HR 9.470, 95% CI 4.092 to 21.918, *P *<* *.001) remained as an independent prognostic biomarker, even after other prognostic biomarkers were controlled for. Loss of CDKL2, however, was not an independent prognostic biomarker (HR 0.705, 95% CI 0.411 to 1.210, *P *=* *.205) (Table 3).

**Table 3:** Univariate and multivariate analyses of prognostic biomarkers in 151 patients with GC^a^

Variables	Univariate		Multivariate	
	HR (95% CI)	*P* b	HR (95% CI)	*P* b
CDKL2				
Low expression	0.508 (0.310‐0.833)	.007	0.705 (0.411‐1.210)	.205
High expression				
Age of patients (y)				
≥66	1.104 (0.674‐1.806)	.695	—	—
<66				
Gender of patients				
Male	0.755 (0.452‐1.260)	.281	—	—
Female				
Lauren classification				
Intestinal	1.743 (1.082‐2.807)	.022	0.697 (0.333‐1.461)	.339
Diffuse				
Invasive depth of tumor				
T1 + T2	3.627 (1.655‐7.944)	.001	1.247 (0.494‐3.152)	.641
T3 + T4				
Regional lymph node metastasis				
N0	5.156 (2.455‐10.831)	<.001	1.661 (0.596‐4.631)	.332
N1 + N2 + N3				
Distant metastasis				
Absent	17.096 (7.517‐38.883)	<.001	10.205 (4.379‐23.779)	<.001
Present				
Pathologic staging				
I + II	5.627 (3.004‐10.540)	<.001	2.872 (1.077‐7.656)	.035
III + IV				
Histologic type and grade				
Poor	0.468 (0.291‐0.753)	.002	0.537 (0.248‐1.163)	.115
Well to moderate				
Lymphovascular permeation				
Absent	3.621 (1.842‐7.115)	<.001	1.137 (0.509‐2.539)	.754
Present				
Adjuvant chemotherapy				
Absent	3.986 (2.358‐6.739)	<.001	1.465 (0.671‐3.194)	.338
Present				

HR, hazard ratio; CI, confidence interval.

a This table shows disease‐free survival.

b All statistical tests were two‐tailed. Significance level: *P *<* *.05.

### 3.4 CDKL2 overexpression inhibited cell proliferation and invasion in GC cells

**Figure 3:**
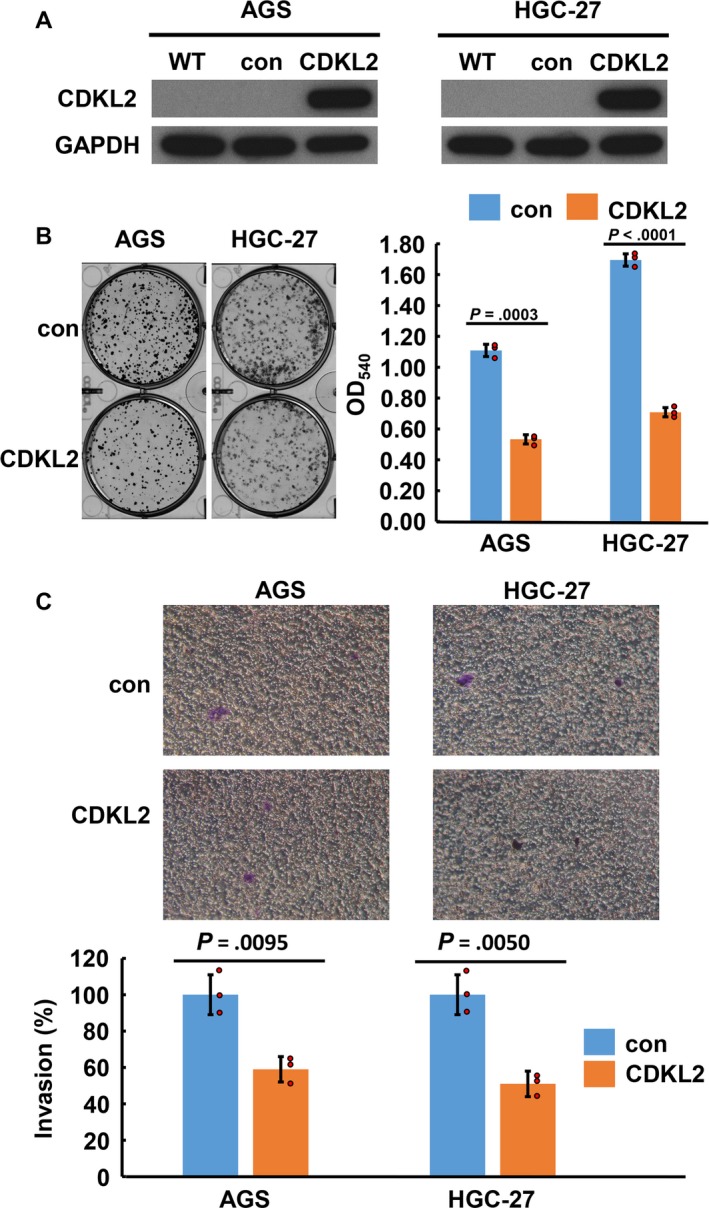
Verification of CDKL2 overexpression in AGS and HGC‐27 cells and the effect of CDKL2 overexpression on cell proliferation and invasion. The immunoblotting results (A) indicate that CDKL2 was efficiently overexpressed by transfection. The blots were first hybridized with CDKL2 antibody and, after stripping, rehybridized with β‐actin antibody. The immunoblots in the figure were cropped. B, CDKL2 overexpression suppressed cell proliferation. The histogram represents OD_540_ (presented as mean ± SD). The assay was performed 3 times. Red circles were used to show individual values. Significance level: *P *<* *.05. C, CDKL2 overexpression repressed cell invasion. The histogram represents cell invasion (presented as mean ± SD). The assay was performed 3 times. Red circles were used to show individual values. Significance level: *P *<* *.05. The differences in cell proliferation and invasion between CDKL2 overexpressed and control cells were analyzed using Student’s *t* test. Significance level: *P *<* *.05

To determine the effect of CDKL2 overexpression on cell proliferation, 2 GC cell lines with low CDKL2 levels—AGS and HGC‐27 cells—were transfected with CDKL2 cDNA ORF vectors to generate CDKL2‐overexpressed cells (Figures 1D and 3A). According to our results, compared with control vectors, the proliferative abilities of AGS and HGC‐27 cells transfected with CDKL2 cDNA ORF vectors were significantly impaired (Figure 3B). Finally, the role of CDKL2 in the invasiveness of the AGS and HGC‐27 cells was investigated. We also found that cell invasion was significantly inhibited in the CDKL2‐overexpressed cells than in the control cells (Figure 3C). These results indicate that CDKL2 overexpression hinders GC cell proliferation and invasion in vitro.

## 4 DISCUSSION

Similar to most cancers, GC has a molecular genetic basis that depends on the abnormalities in normal cellular regulatory mechanisms that govern cell proliferation.^26^ In this study, we measured CDKL2 expression in GC and analyzed the relationship between CDKL2 expression and different clinical and pathological characteristics. Our results reveal a significant downregulation of the CDKL2 protein in human GC cells and tissues, and the decreased CDKL2 level was positively correlated with Lauren classification, pathologic staging, histologic type and grade, and short patient survival. Furthermore, CDKL2 downregulation is an unfavorable prognosticator for GC, and forced CDKL2 expression in human GC cell lines hindered cell proliferation and impaired invasiveness.

Studies on CDKL expression in various cancers are scarce and controversial. Jones et al described a gene signature in which CDKL1 was downregulated in renal cell cancer, but Qin et al revealed that CDKL1 was upregulated in colorectal cancer.^27^, ^28^ Kawahara and colleagues demonstrated that CDKL5 was overexpressed in leukemia cells but not in normal T cells.^29^ To date, only one study performed by Li and colleagues showed the expression of CDKL2 and indicated that CDKL2 was considerably overexpressed in human breast cancer tissues and cells compared with normal breast tissues and cells.^23^ By contrast, our data show the loss of CDKL2 in GC. Our data are in line with other data described in the Oncomine database. One reason for the discrepancy between our study and Li’s study may result from the different molecules examined. In our study, CDKL2 protein was measured, and in another, CDKL2 mRNA was detected. Another explanation for the discrepancy is that the CDKL2 expression is cell context‐specific. However, notably, even in breast cancer, the data from Li’s study are different from those described in the Oncomine database. The reason for the discrepancy between Li’s study and the Oncomine database may result from the different sample size. Overall, these studies suggested that the expression of CDKLs in human cancers seems to be more complicated than expected and warrants additional studies. This is the first study to report the expression of CDKL2 in GC.

**Figure 4:**
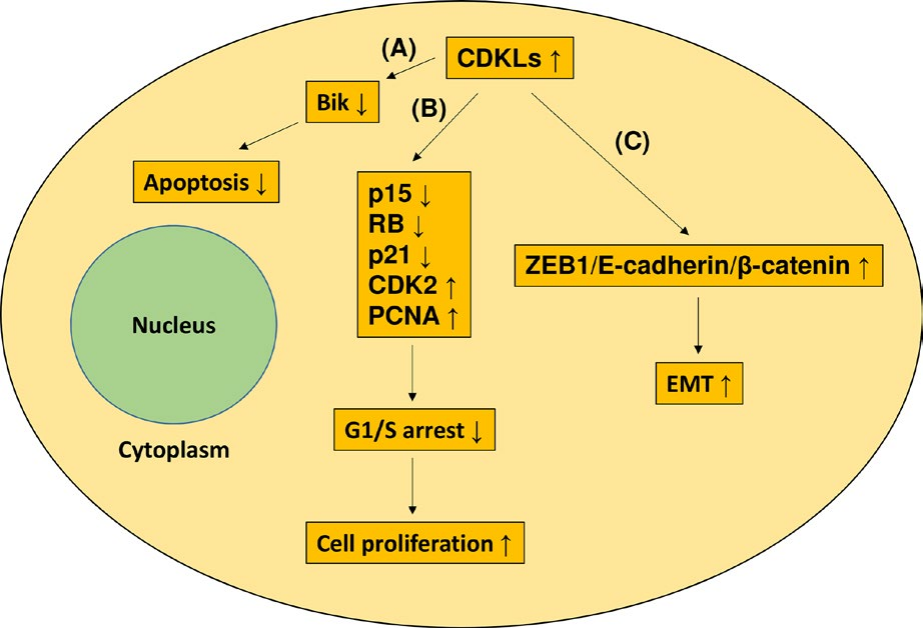
Cellular effects of CDKL overexpression in GC. CDKL overexpression can elicit 3 possible effects. A, CDKL overexpression can inhibit apoptosis through inactivating the Bik pro‐apoptotic protein. B, CDKL overexpression can downregulate the p15, RB, and p21 expression, upregulate CDK2 and PCNA expression, and then, promote G1‐S transition and cell proliferation. C, CDKL2 can facilitate EMT through activating a positive feedback loop comprising ZEB1, E‐cadherin, and β‐catenin

The CDK family is crucial in the regulation of cell cycle progression at the G1/S and G2/M checkpoints.^30^ The CDKL family, which is considered separate from the CDK family, was recently identified through biochemical and genetic approaches.^13^ The role that CDKLs play in cancer is not fully understood. Several studies have shown that CDKLs were potential oncogenes and had roles in tumor development, and these results are summarized in Figure 4. For instance, cell growth, tumor invasion, and cell cycle progression of colorectal cells were considerably hindered through CDKL1 silencing.^28^ CDKL1 was also revealed to downregulate the expression of p15 and RB and then promote G1‐S transition. Similar results were also observed in melanoma: Suppression of CDKL1 in melanoma cells considerably delayed cell growth, induced cell apoptosis, and stopped cell cycle progression at the G1 phase.^31^ Furthermore, CDKL1 was shown to facilitate cell cycle progression through decreasing the expression of p21 and increasing the expression of CDK2. One study conducted on GC demonstrated that CDKL1 knockdown decreased cellular proliferation and increased apoptosis.^25^ Moreover, CDKL1 was shown to inhibit the activation of Bik pro‐apoptotic protein and enhance the expression of PCNA. CDKL2‐expressing human mammary epithelial cells enhanced EMT and stem cell properties. In addition, CDKL2 promoted xenograft proliferation and metastasis in vivo.^23^ CDKL2 was reported to facilitate EMT by activating a positive feedback loop comprising ZEB1, E‐cadherin, and β‐catenin. However, in this study, CDKL2 functioned as a tumor suppressor and enforced expression of CDKL2 inhibited GC cell proliferation and invasion. According to the publicly available Oncomine database, the CDKL2 level in nontumor tissues is higher than that in tumor tissues in several cancers. The mechanisms that make CDKLs to be tumor suppressors have not been reported. These mechanisms must be elucidated in additional studies.

Few studies exist to suggest the effects of CDKLs on tumor prognosis. A study demonstrated that an increased copy number of *cdkl4* in colorectal cancer was predictive of poorer patient survival.^32^ Varghese’s group reported that CDKL5 overexpression was associated with poor prognosis for patients with glioblastoma.^33^ The only study conducted on breast cancer showed that patients with upregulated CDKL2 expression had a significantly poorer survival rate compared with patients without this change.^23^ However, our results indicate that loss of CDKL2 was negatively correlated with patient survival. In accordance with the aforementioned explanation of the expression profiles of CDKL2 in breast cancer and GC, the reason for the discrepancy may result from the different molecules examined. Furthermore, multivariate Cox regression analysis revealed that loss of CDKL2 was not an independent prognostic biomarker. It suggested that loss of CDKL2 in GC can be a useful prognostic predictor in conjunction with other conventional important prognostic factors such as pathologic stage and distant metastasis. A group of advanced GC patients with low CDKL2 expression should be considered for aggressive treatment and be clinically evaluated and followed up more closely. This is the first study to reveal that loss of CDKL2 is a prognostic biomarker for GC.

In conclusion, our findings indicate that loss of CDKL2 promotes a malignant phenotype of GC and illustrate the clinical significance of loss of CDKL2 in GC.

## CONFLICT OF INTEREST

The authors have no conflict of interest to declare.
