# Supplementary material for: Loss of cyclin‐dependent kinase‐like 2 predicts poor prognosis in gastric cancer, and its overexpression suppresses cells growth and invasion
Source: Cancer Med. 2018 May 23;7(7):2993–3002. doi: 10.1002/cam4.1577 (PMC6051174; doi:10.1002/cam4.1577)

The uncropped whole-membrane photograph of immunoblot showing the expression of CDKL2 in 5 gastric cell lines and two non-tumor/tumor pairs of gastric tissues.

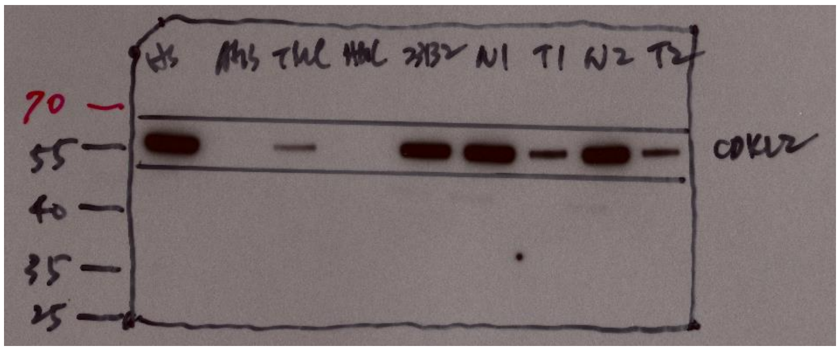

The uncropped whole-membrane photograph of immunoblot showing the expression of GAPDH in 5 gastric cell lines and two non-tumor/tumor pairs of gastric tissues.

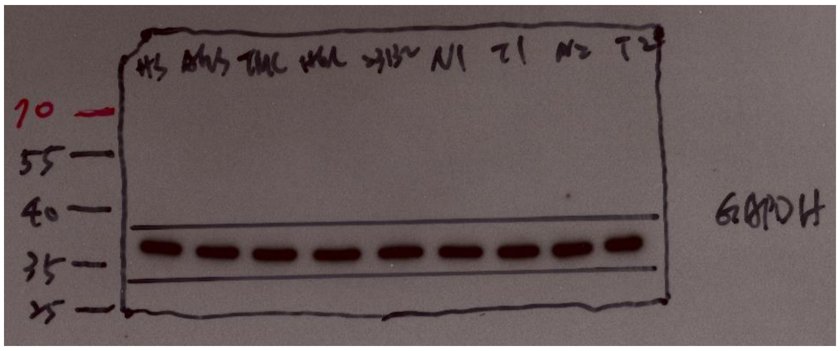

The uncropped whole-membrane photograph of immunoblot showing the expression of CDKL2 in CDKL2-manipulated AGS and HGC-27 cells.

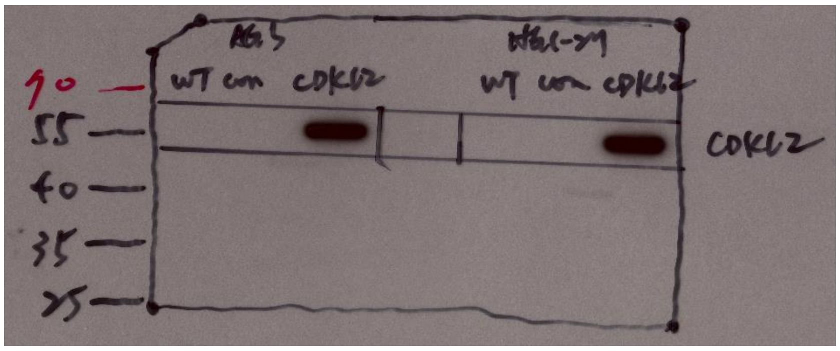

The uncropped whole-membrane photograph of immunoblot showing the expression of CDKL2 in CDKL2-manipulated AGS and HGC-27 cells.

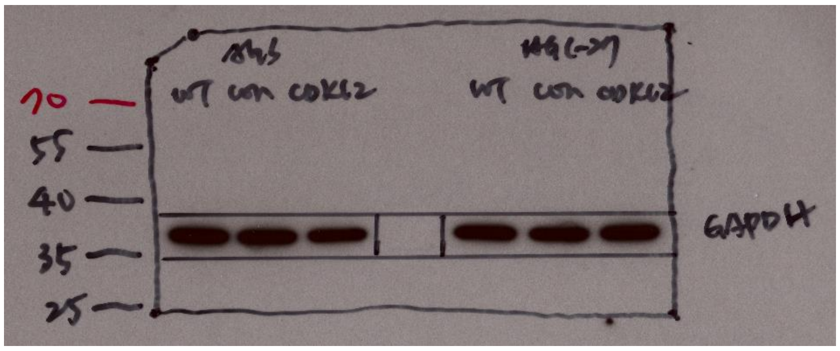

Supplement: Supplementary file 1 [file CAM4-7-2993-s001.pdf]
